# State Medicaid Coverage for Tobacco Cessation Treatments and Barriers to Accessing Treatments — United States, 2015–2017

**DOI:** 10.15585/mmwr.mm6713a3

**Published:** 2018-04-06

**Authors:** Anne DiGiulio, Zach Jump, Annie Yu, Stephen Babb, Anna Schecter, Kisha-Ann S. Williams, Debbie Yembra, Brian S. Armour

**Affiliations:** ^1^American Lung Association, Chicago, Illinois; ^2^Office on Smoking and Health, National Center for Chronic Disease Prevention and Health Promotion, CDC.

Cigarette smoking prevalence among Medicaid enrollees (25.3%) is approximately twice that of privately insured Americans (11.8%), placing Medicaid enrollees at increased risk for smoking-related disease and death (*1*). Medicaid spends approximately $39 billion annually on treating smoking-related diseases (*2*). Individual, group, and telephone counseling and seven Food and Drug Administration (FDA)–approved medications* are effective in helping tobacco users quit (*3*). Although state Medicaid coverage of tobacco cessation treatments improved during 2014–2015, coverage was still limited in most states (*4*). To monitor recent changes in state Medicaid cessation coverage for traditional (i.e., nonexpansion) Medicaid enrollees, the American Lung Association collected data on coverage of a total of nine cessation treatments: individual counseling, group counseling, and seven FDA-approved cessation medications^†^ in state Medicaid programs during July 1, 2015–June 30, 2017. The American Lung Association also collected data on seven barriers to accessing covered treatments, such as copayments and prior authorization. As of June 30, 2017, 10 states covered all nine of these treatments for all enrollees, up from nine states as of June 30, 2015; of these 10 states, Missouri was the only state to have removed all seven barriers to accessing these cessation treatments. State Medicaid programs that cover all evidence-based cessation treatments, remove barriers to accessing these treatments, and promote covered treatments to Medicaid enrollees and health care providers would be expected to reduce smoking, smoking-related disease, and smoking-attributable federal and state health care expenditures (*5*–*7*).

During July 2015–June 2017, the American Lung Association compiled data on state Medicaid tobacco cessation coverage from state Medicaid and Medicaid managed care plan member and provider websites and handbooks, policy manuals, plan formularies and preferred drug lists, Medicaid state plan amendments, and relevant regulations and laws.^§^ Analysts searched for mentions of the nine cessation treatments using search functions on state Medicaid websites and other relevant state-sponsored websites and the Google search engine. The American Lung Association contacted personnel from state Medicaid agencies, state health departments, or other state government agencies to give them the opportunity to verify the information collected and to retrieve missing documents and reconcile discrepancies.

As of June 30, 2017, 10 states (California, Connecticut, Indiana, Maine, Massachusetts, Minnesota, Missouri, New York, Ohio, and Vermont) covered all nine cessation treatments for all Medicaid enrollees, an increase from nine states in June 2015 (Table 1) (Table 2). Three states (California, Missouri, and New York) achieved this level of coverage during the study period. Conversely, North Dakota and Pennsylvania, which covered all nine cessation treatments in June 2015, no longer did so in June 2017.^¶^ As of June 30, 2017, nine of the 10 states that covered all cessation treatments had barriers in place for some treatments (Table 3); the remaining state, Missouri, has removed all barriers examined in this study. Two additional states (Kentucky and South Carolina) achieved comprehensive coverage effective July 1, 2017, after conclusion of the study period; Kentucky also removed all barriers to accessing the nine cessation treatments.**

**TABLE 1:** Medicaid coverage for tobacco cessation counseling, by state — United States, 2015 and 2017*^,†^

State	Individual counseling		Group counseling	
	2015	2017	2015	2017
Alabama	P	P	No	No
Alaska	Yes	Yes	No	No
Arizona	P	P	No	No
Arkansas	Yes	Yes	No	No
California	V	Yes	V	Yes
Colorado	P	Yes	P	V
Connecticut	Yes	Yes	Yes	Yes
Delaware	Yes	Yes	No	No
District of Columbia	NA	Yes	NA	V
Florida	V	V	V	V
Georgia	Yes	Yes	No	No
Hawaii	V	Yes	V	V
Idaho	Yes	Yes	No	No
Illinois	No	No	No	No
Indiana	Yes	Yes	Yes	Yes
Iowa	Yes	Yes	No	V
Kansas	P	P	P	P
Kentucky	V	V	V	V
Louisiana	No	No	V	V
Maine	Yes	Yes	Yes	Yes
Maryland	Yes	Yes	V	V
Massachusetts	Yes	Yes	Yes	Yes
Michigan	Yes	Yes	V	V
Minnesota	Yes	Yes	Yes	Yes
Mississippi	V	V	No	V
Missouri	Yes	Yes	No	Yes
Montana	Yes	Yes	No	No
Nebraska	Yes	Yes	No	No
Nevada	Yes	V	No	V
New Hampshire	Yes	Yes	V	V
New Jersey	Yes	V	No	No
New Mexico	Yes	V	No	V
New York	Yes	Yes	Yes	Yes
North Carolina	Yes	Yes	No	No
North Dakota	Yes	No	Yes	No
Ohio	Yes	Yes	Yes	Yes
Oklahoma	Yes	Yes	No	No
Oregon	Yes	Yes	V	V
Pennsylvania	Yes	Yes	Yes	V
Rhode Island	Yes	Yes	V	V
South Carolina	V	V	V	V
South Dakota	P	P	No	No
Tennessee	No	P	No	No
Texas	V	V	V	V
Utah	P	Yes	P	P
Vermont	Yes	Yes	Yes	Yes
Virginia	V	V	V	V
Washington	V	V	No	No
West Virginia	No	Yes	V	No
Wisconsin	Yes	Yes	V	V
Wyoming	Yes	Yes	No	No
**Total count**				
**Yes**	**31**	**33**	**10**	**10**
**No**	**4**	**3**	**22**	**19**
**V**	**9**	**10**	**15**	**20**
**P**	**6**	**5**	**3**	**2**
**NA**	**1**	**0**	**1**	**0**

**Abbreviations:** NA = information not available; No = treatment not covered for any Medicaid enrollee; P = treatment covered for pregnant women only; V = coverage varies, with treatment covered for some, but not all, Medicaid enrollees; Yes = treatment covered for all Medicaid enrollees.

* Data as of June 30, 2015, and June 30, 2017.

^†^ Because of differences in the methods and timing of data collection, some findings differ from findings on this topic reported in *MMWR* before 2014 (https://www.cdc.gov/mmwr/preview/mmwrhtml/mm5941a4.htm).

**TABLE 2:** Medicaid coverage for tobacco cessation medications, by state — United States, 2015 and 2017*^,†^

State	NRT patch		NRT gum		NRT lozenge		NRT nasal spray		NRT inhaler		Bupropion (Zyban)		Varenicline (Chantix)	
	2015	2017	2015	2017	2015	2017	2015	2017	2015	2017	2015	2017	2015	2017
Alabama	Yes	Yes	Yes	Yes	Yes	Yes	Yes	Yes	Yes	Yes	Yes	Yes	Yes	Yes
Alaska	Yes	Yes	Yes	Yes	Yes	Yes	Yes	Yes	Yes	Yes	Yes	Yes	Yes	Yes
Arizona	Yes	Yes	Yes	Yes	Yes	Yes	Yes	Yes	Yes	Yes	Yes	Yes	Yes	Yes
Arkansas	Yes	Yes	Yes	Yes	No	No	No	No	No	No	Yes	Yes	Yes	Yes
California	Yes	Yes	Yes	Yes	Yes	Yes	Yes	Yes	Yes	Yes	Yes	Yes	Yes	Yes
Colorado	Yes	Yes	Yes	Yes	Yes	Yes	Yes	Yes	Yes	Yes	Yes	Yes	Yes	Yes
Connecticut	Yes	Yes	Yes	Yes	Yes	Yes	Yes	Yes	Yes	Yes	Yes	Yes	Yes	Yes
Delaware	Yes	Yes	Yes	Yes	Yes	Yes	Yes	Yes	Yes	Yes	Yes	Yes	Yes	Yes
District of Columbia	NA	Yes	NA	Yes	NA	Yes	NA	V	NA	V	NA	Yes	NA	Yes
Florida	Yes	V	Yes	V	Yes	V	No	No	No	No	Yes	Yes	Yes	Yes
Georgia	Yes	Yes	Yes	Yes	Yes	Yes	Yes	V	Yes	V	Yes	Yes	Yes	V
Hawaii	Yes	Yes	Yes	Yes	V	V	V	V	V	V	V	Yes	V	Yes
Idaho	Yes	Yes	Yes	Yes	Yes	Yes	Yes	Yes	Yes	Yes	Yes	Yes	Yes	Yes
Illinois	Yes	Yes	Yes	Yes	Yes	Yes	Yes	Yes	Yes	Yes	Yes	Yes	Yes	Yes
Indiana	Yes	Yes	Yes	Yes	Yes	Yes	Yes	Yes	Yes	Yes	Yes	Yes	Yes	Yes
Iowa	Yes	Yes	Yes	Yes	Yes	Yes	Yes	Yes	Yes	Yes	Yes	Yes	Yes	Yes
Kansas	Yes	Yes	Yes	Yes	Yes	Yes	Yes	Yes	Yes	Yes	Yes	Yes	Yes	Yes
Kentucky	Yes	Yes	V	Yes	V	Yes	V	Yes	V	Yes	V	Yes	V	Yes
Louisiana	V	V	V	V	V	V	V	V	V	V	Yes	Yes	V	V
Maine	Yes	Yes	Yes	Yes	Yes	Yes	Yes	Yes	Yes	Yes	Yes	Yes	Yes	Yes
Maryland	Yes	Yes	Yes	Yes	Yes	Yes	Yes	Yes	Yes	Yes	Yes	Yes	Yes	Yes
Massachusetts	Yes	Yes	Yes	Yes	Yes	Yes	Yes	Yes	Yes	Yes	Yes	Yes	Yes	Yes
Michigan	Yes	Yes	Yes	Yes	Yes	Yes	V	Yes	Yes	Yes	Yes	Yes	Yes	Yes
Minnesota	Yes	Yes	Yes	Yes	Yes	Yes	Yes	Yes	Yes	Yes	Yes	Yes	Yes	Yes
Mississippi	Yes	Yes	Yes	Yes	Yes	Yes	V	Yes	V	Yes	Yes	Yes	Yes	Yes
Missouri	Yes	Yes	Yes	Yes	Yes	Yes	Yes	Yes	Yes	Yes	Yes	Yes	Yes	Yes
Montana	Yes	Yes	Yes	Yes	Yes	Yes	No	No	No	Yes	Yes	Yes	Yes	Yes
Nebraska	Yes	Yes	Yes	Yes	Yes	Yes	Yes	Yes	Yes	Yes	Yes	Yes	Yes	Yes
Nevada	Yes	V	Yes	V	Yes	V	Yes	Yes	Yes	Yes	Yes	Yes	Yes	Yes
New Hampshire	Yes	Yes	Yes	Yes	Yes	Yes	Yes	Yes	Yes	Yes	Yes	Yes	Yes	Yes
New Jersey	Yes	Yes	Yes	V	Yes	V	Yes	V	Yes	V	Yes	Yes	Yes	Yes
New Mexico	Yes	Yes	Yes	Yes	Yes	Yes	V	V	V	V	V	Yes	Yes	Yes
New York	Yes	Yes	Yes	Yes	V	Yes	V	Yes	V	Yes	Yes	Yes	Yes	Yes
North Carolina	Yes	Yes	Yes	Yes	Yes	Yes	Yes	Yes	Yes	Yes	Yes	Yes	Yes	Yes
North Dakota	Yes	Yes	Yes	Yes	Yes	Yes	Yes	Yes	Yes	Yes	Yes	Yes	Yes	Yes
Ohio	Yes	Yes	Yes	Yes	Yes	Yes	Yes	Yes	Yes	Yes	Yes	Yes	Yes	Yes
Oklahoma	Yes	Yes	Yes	Yes	Yes	Yes	Yes	Yes	Yes	Yes	Yes	Yes	Yes	Yes
Oregon	V	Yes	V	Yes	V	V	V	V	V	V	V	Yes	V	Yes
Pennsylvania	Yes	Yes	Yes	Yes	Yes	Yes	Yes	V	Yes	V	Yes	Yes	Yes	V
Rhode Island	Yes	Yes	Yes	Yes	Yes	V	Yes	No	Yes	No	Yes	Yes	Yes	Yes
South Carolina	Yes	Yes	Yes	Yes	V	V	V	V	V	V	V	Yes	V	V
South Dakota	P	No	P	No	P	No	No	No	No	No	Yes	Yes	Yes	Yes
Tennessee	Yes	Yes	Yes	Yes	Yes	Yes	Yes	Yes	Yes	Yes	Yes	Yes	Yes	Yes
Texas	Yes	Yes	Yes	Yes	Yes	Yes	No	Yes	No	Yes	Yes	Yes	Yes	Yes
Utah	V	V	V	V	V	V	V	V	V	V	Yes	Yes	Yes	Yes
Vermont	Yes	Yes	Yes	Yes	Yes	Yes	Yes	Yes	Yes	Yes	Yes	Yes	Yes	Yes
Virginia	Yes	Yes	V	V	V	V	V	V	V	V	V	V	V	V
Washington	V	Yes	V	Yes	V	V	V	V	V	V	V	Yes	V	V
West Virginia	Yes	Yes	Yes	Yes	Yes	Yes	Yes	Yes	Yes	Yes	Yes	Yes	No	Yes
Wisconsin	Yes	Yes	Yes	Yes	No	Yes	Yes	Yes	Yes	Yes	Yes	Yes	Yes	Yes
Wyoming	Yes	Yes	Yes	Yes	Yes	Yes	No	No	No	No	Yes	Yes	Yes	Yes
**Total count**														
Yes	45	46	43	44	38	38	32	33	33	34	43	50	42	45
No	0	1	0	1	2	2	6	6	6	5	0	0	1	0
V	4	4	6	6	9	11	12	12	11	12	7	1	7	6
P	1	0	1	0	1	0	0	0	0	0	0	0	0	0
NA	1	0	1	0	1	0	1	0	1	0	1	0	1	0

**Abbreviations:** NA = information not available; No = treatment not covered for any Medicaid enrollee; NRT = nicotine replacement therapy; P = treatment covered for pregnant women only; V = coverage varies, with treatment covered for some, but not all, Medicaid enrollees; Yes = treatment covered for all Medicaid enrollees.

* Data as of June 30, 2015, and June 30, 2017.

^†^ Because of differences in the methods and timing of data collection, some findings differ from findings on this topic reported in *MMWR* before 2014 (https://www.cdc.gov/mmwr/preview/mmwrhtml/mm5941a4.htm).

**TABLE 3:** Barriers to Medicaid coverage for tobacco cessation treatments, by state — United States, 2015 and 2017*^,†^

State	Copayments required		Prior authorization required		Counseling required for medications		Stepped-care therapy		Limits on duration		Annual limit on quit attempts		Lifetime limit on quit attempts	
	2015	2017	2015	2017	2015	2017	2015	2017	2015	2017	2015	2017	2015	2017
Alabama	Yes	Yes	Yes	Yes	Yes	Yes	No	No	Yes	Yes	Yes	Yes	No	No
Alaska	Yes	Yes	Yes	Yes	No	No	No	No	Yes	Yes	Yes	Yes	No	No
Arizona	No	No	No	No	No	No	No	No	Yes	Yes	Yes	Yes	No	No
Arkansas	No	No	Yes	Yes	Yes	Yes	No	No	Yes	Yes	Yes	Yes	No	No
California	No	No	V	V	No	No	V	No	V	V	V	V	No	No
Colorado	V	No	Yes	Yes	V	No	No	V	Yes	Yes	Yes	Yes	No	No
Connecticut	No	No	Yes	Yes	No	No	No	No	Yes	Yes	No	No	No	No
Delaware	Yes	Yes	Yes	Yes	Yes	Yes	Yes	Yes	No	Yes	Yes	Yes	No	No
District of Columbia	NA	No	NA	V	NA	No	NA	No	NA	V	NA	V	NA	No
Florida	V	V	V	No	No	No	V	No	V	Yes	V	No	V	No
Georgia	No	No	Yes	V	Yes	Yes	Yes	Yes	Yes	Yes	Yes	Yes	No	No
Hawaii	V	No	V	V	V	V	V	V	V	V	Yes	Yes	No	No
Idaho	No	No	Yes	Yes	No	Yes	Yes	Yes	Yes	Yes	Yes	Yes	No	No
Illinois	Yes	Yes	No	No	No	No	No	No	No	No	No	No	No	No
Indiana	Yes	Yes	No	No	Yes	Yes	Yes	No	Yes	Yes	Yes	Yes	No	No
Iowa	Yes	No	Yes	Yes	Yes	Yes	Yes	Yes	Yes	Yes	Yes	Yes	No	No
Kansas	No	No	No	No	No	No	No	No	Yes	Yes	Yes	Yes	No	No
Kentucky	V	No	V	V	V	No	No	No	V	Yes	V	Yes	No	No
Louisiana	Yes	Yes	V	V	V	V	No	No	V	V	No	No	No	No
Maine	No	No	Yes	Yes	No	No	Yes	Yes	No	No	No	No	No	No
Maryland	V	No	V	Yes	V	No	V	Yes	V	Yes	V	Yes	No	No
Massachusetts	Yes	Yes	Yes	Yes	No	No	No	No	No	No	Yes	Yes	No	No
Michigan	No	No	V	No	No	No	V	No	V	V	Yes	No	No	No
Minnesota	Yes	No	No	V	No	No	No	No	No	V	No	No	No	No
Mississippi	V	V	No	No	No	No	No	Yes	Yes	Yes	No	No	No	No
Missouri	No	No	Yes	No	No	No	No	No	Yes	No	No	No	Yes	No
Montana	No	No	Yes	Yes	No	No	Yes	Yes	Yes	Yes	Yes	Yes	No	No
Nebraska	Yes	Yes	Yes	Yes	Yes	Yes	No	No	Yes	Yes	Yes	Yes	No	No
Nevada	Yes	No	Yes	Yes	No	No	No	V	Yes	Yes	Yes	Yes	No	No
New Hampshire	Yes	V	Yes	V	No	No	No	No	No	V	Yes	V	No	No
New Jersey	V	No	V	V	No	No	No	V	V	V	V	V	V	V
New Mexico	No	V	V	V	V	V	No	No	V	V	V	V	No	No
New York	V	V	V	No	No	No	No	No	Yes	No	Yes	No	No	No
North Carolina	Yes	Yes	Yes	Yes	No	No	Yes	Yes	Yes	Yes	No	No	No	No
North Dakota	Yes	Yes	Yes	Yes	Yes	Yes	No	No	Yes	Yes	Yes	Yes	No	No
Ohio	Yes	No	V	V	No	No	V	V	No	V	No	V	No	No
Oklahoma	Yes	No	No	No	No	No	No	No	Yes	Yes	Yes	Yes	No	No
Oregon	No	No	V	V	V	V	No	V	V	V	V	V	No	No
Pennsylvania	Yes	V	V	V	No	No	No	No	Yes	Yes	No	No	No	No
Rhode Island	No	No	Yes	Yes	Yes	No	Yes	No	Yes	V	Yes	No	No	No
South Carolina	V	V	V	V	V	V	V	V	Yes	Yes	V	V	No	No
South Dakota	Yes	Yes	No	No	No	No	No	No	No	No	No	No	No	No
Tennessee	No	No	Yes	Yes	No	No	Yes	Yes	No	No	Yes	Yes	No	No
Texas	Yes	Yes	No	Yes	No	No	No	No	No	No	No	No	No	No
Utah	Yes	Yes	Yes	Yes	No	No	No	No	No	No	No	No	No	No
Vermont	Yes	Yes	Yes	Yes	No	No	No	Yes	Yes	Yes	Yes	Yes	No	No
Virginia	V	V	V	V	V	V	V	V	V	V	V	V	No	No
Washington	No	No	V	V	V	V	No	No	V	V	V	V	V	V
West Virginia	Yes	No	Yes	Yes	Yes	No	Yes	No	Yes	Yes	Yes	Yes	No	No
Wisconsin	Yes	Yes	No	No	No	No	No	No	No	No	No	No	No	No
Wyoming	Yes	Yes	No	No	No	No	No	No	Yes	Yes	Yes	Yes	No	No
**Total count**														
Yes	24	16	23	22	10	9	11	11	26	27	26	24	1	0
No	16	27	11	13	30	35	31	32	12	10	14	17	46	49
V	10	8	16	16	10	7	8	8	12	14	10	10	3	2
NA	1	0	1	0	1	0	1	0	1	0	1	0	1	0

**Abbreviations:** NA = information not available; No = barrier does not apply to any Medicaid enrollee for any treatment; V = varies, barrier applies to some, but not all, Medicaid enrollees for one or more treatments; Yes = barrier applies to all Medicaid enrollees for one or more treatments.

* Data as of June 30, 2015, and June 30, 2017.

^†^ Because of differences in the methods and timing of data collection, some findings differ from findings on this topic reported in *MMWR* before 2014 (https://www.cdc.gov/mmwr/preview/mmwrhtml/mm5941a4.htm).

As of June 30, 2017, all 50 states and the District of Columbia (DC) covered at least some cessation treatments for all Medicaid enrollees, compared with 48 states in June 2015. As of June 30, 2017, 32 states covered all seven FDA-approved cessation medications for all enrollees, up from 30 states in June 2015 (Table 2). Thirty-three states covered individual counseling as of June 30, 2017, with 10 of these states covering group counseling as well, compared with 31 states and 10 states, respectively, as of June 2015 (Table 1).

During July 1, 2015–June 30, 2017, 13 states removed copayments for cessation treatments for at least some Medicaid enrollees, and the number of states that do not require copayments for any cessation treatment for any Medicaid enrollees increased from 16 to 27 states. As of June 30, 2017, the most common barriers were limits on duration (with 41 states reporting this barrier for at least certain populations or plans), prior authorization requirements (38 states), annual limits on quit attempts (34 states), and required copayments (24 states) (Table 3). 

## Discussion

Some progress occurred in state Medicaid coverage of proven tobacco cessation treatments during July 2015–June 2017, with the number of states covering all nine cessation treatments for all traditional (i.e., nonexpansion) Medicaid enrollees increasing from nine to 10 and the number of states covering all seven FDA-approved cessation medications increasing from 30 to 32. However, coverage still falls substantially short of the *Healthy People 2020* objective of comprehensive cessation coverage in all 50 states and DC.^††^ Moreover, as of June 2017, all but one state retained barriers that make it more difficult for Medicaid enrollees to access cessation treatments. Removing these barriers would be expected to increase access to and use of cessation treatments (*3*,*6*). Comprehensive Medicaid tobacco cessation coverage with minimal barriers can help more Medicaid enrollees quit smoking, resulting in improved health and potentially reducing smoking-attributable Medicaid expenditures (*5*–*7*).

The increase in the number of states covering all seven FDA-approved cessation medications might have resulted, in part, from a federal requirement that traditional state Medicaid programs cover these medications (*8*).^§§^ State Medicaid programs can maximize the impact of this coverage by placing cessation medications on preferred drug lists, removing barriers to access, and adding notices of coverage to public plan documents. In 2015, 69.2% of adult Medicaid enrollees nationally who smoked reported wanting to quit, and 56.3% had made a quit attempt in the past year (*9*). However, only 34.5% of adult smokers on Medicaid used cessation counseling, medication, or both when trying to quit (with 8.0% using counseling and 32.2% using medication); 59.9% who had seen a health professional in the past year received advice to quit, and 5.9% succeeded in quitting (*9*). Comprehensive coverage of evidence-based cessation treatments could increase quit attempts, use of cessation treatments, and quit rates (*3*,*5**,**6*).

These findings indicate that state Medicaid coverage of tobacco cessation counseling is lagging behind coverage of cessation medications. State Medicaid programs can increase tobacco cessation among Medicaid enrollees by covering cessation counseling along with cessation medications; the combined use of these treatments is more effective in increasing quit rates than is the use of either treatment alone (*3*). However, requiring Medicaid enrollees to obtain counseling as a precondition for receiving medications has the potential to decrease enrollees’ use of medications (*10*). State Medicaid programs can further increase the number of enrollees who quit smoking by promoting covered treatments to Medicaid enrollees and their health care providers to increase use of these treatments.

The findings in this report are subject to at least three limitations. First, in cases where official documents were not publicly available or were outdated or conflicting, American Lung Association personnel consulted state government personnel for clarification; the information they provided might have been inaccurate in some cases. Second, cessation coverage can vary widely across Medicaid managed care plans, and these plans and their cessation coverage can change over time, making it challenging to determine state Medicaid managed care plan cessation coverage. Finally, this report does not assess promotion, awareness, or use of state Medicaid cessation coverage.

Approximately 7.9 million adult smokers are estimated to be enrolled in Medicaid (*1*).^¶¶^ The disproportionately high cigarette smoking prevalence among Medicaid enrollees imposes a substantial health burden on society and is a major driver of federal and state health care expenditures. Smoking-related diseases accounted for approximately 15% of annual Medicaid spending during 2006–2010, amounting to approximately $39 billion in 2010 (*2*). State Medicaid programs can maximize tobacco cessation among Medicaid enrollees, which would be expected to reduce this health and financial burden by covering all evidence-based cessation treatments, removing barriers that impede access to these treatments, promoting covered treatments to Medicaid enrollees and their health care providers to increase use of these treatments, and monitoring use of covered treatments (*5*–*7*).

**Summary**

**What is already known about this topic?**

Medicaid enrollees smoke cigarettes at a higher rate than do privately insured U.S. residents (25.3% versus 11.8%). Comprehensive state Medicaid cessation coverage has the potential to reduce smoking, smoking-related disease, and health care expenditures among Medicaid enrollees. A *Healthy People 2020* objective calls for comprehensive tobacco cessation treatment coverage in all 50 states and the District of Columbia.

**What is added by this report?**

Although progress occurred in state Medicaid tobacco cessation coverage during 2015–2017, coverage continues to fall short of the target set by the *Healthy People 2020* objective. As of June 30, 2017, 10 states covered all nine evidence-based cessation treatments considered in this study for all Medicaid enrollees, up from nine states in 2015. All but one of these 10 states had barriers to accessing some treatments. As of June 30, 2017, 32 states covered all seven FDA-approved cessation medications, and 33 states covered individual cessation counseling, with 10 of the latter states also covering group counseling.

**What are the implications for public health practice?**

State Medicaid programs can help Medicaid enrollees quit smoking by covering all evidence-based cessation treatments, removing barriers that make it difficult for enrollees to access these treatments, and promoting covered treatments to increase their use.

## Footnotes

* These medications include the nicotine patch, gum, lozenge, nasal spray, and inhaler and bupropion and varenicline.

† Telephone counseling is available free to callers to state quitlines (including Medicaid enrollees) in all 50 states and the District of Columbia through the national quitline portal 1-800-QUIT-NOW, and therefore is not captured by this report. In June 2011, the Centers for Medicare & Medicaid Services announced that it would offer a 50% federal administrative match to state Medicaid programs for the cost of state quitline counseling provided to Medicaid enrollees.

§ Information on state Medicaid cessation coverage compiled by the American Lung Association is available in the CDC State Activities Tracking and Evaluation (STATE) System, a database that contains tobacco-related epidemiologic and economic data and information on state tobacco-related legislation (https://www.cdc.gov/statesystem). Certain data presented in this report differ slightly from Medicaid cessation coverage data reported in the STATE System because of small differences in coding rules, categories, and reporting periods.

¶ These two states are no longer considered to provide comprehensive Medicaid cessation coverage because of a change in how cessation counseling benefits are administered in North Dakota and the addition of a new Medicaid managed care plan in Pennsylvania that did not provide comprehensive coverage.

** Kentucky achieved comprehensive Medicaid cessation coverage and removed barriers impeding Medicaid enrollees’ access to cessation treatments by enacting a state law (Ky. Rev. Stat. Ann. Sect. 205) that also applied to private cessation insurance coverage. South Carolina achieved comprehensive Medicaid cessation coverage by issuing a Medicaid bulletin (https://www.scdhhs.gov/press-release/tobacco-cessation-coverage).

†† https://www.healthypeople.gov/2020/topics-objectives/topic/tobacco-use/objectives.

§§ Effective January 2014, section 2502 of the 2010 Patient Protection and Affordable Care Act barred state Medicaid programs from excluding FDA-approved cessation medications from coverage: Patient Protection and Affordable Care Act of 2010. Pub. L. No. 114–48 (March 23, 2010, as amended through May 1, 2010 (http://housedocs.house.gov/energycommerce/ppacacon.pdf ). The Centers for Medicare & Medicaid Services has issued guidance to states on implementing this provision (https://www.medicaid.gov/Medicaid-CHIP-Program-Information/By-Topics/Prescription-Drugs/Downloads/Rx-Releases/State-Releases/state-rel-165.pdf). As of June 30, 2017, the Centers for Medicare & Medicaid Services had published state plan amendments from 37 states declaring that they have implemented this provision.

¶¶ This estimate includes both traditional and expansion Medicaid enrollees.
